# Constructing Reservoir Area–Volume–Elevation Curve from TanDEM-X DEM Data

**DOI:** 10.1109/jstars.2021.3051103

**Published:** 2021-01-12

**Authors:** Yao Li, Huilin Gao, George H. Allen, Zhe Zhang

**Affiliations:** Zachry Department of Civil and Environmental Engineering, Texas A&M University, College Station, TX 77843 USA; Zachry Department of Civil and Environmental Engineering, Texas A&M University, College Station, TX 77843 USA; Department of Geography, Texas A&M University, College Station, TX77843 USA; Department of Geography, Texas A&M University, College Station, TX77843 USA

**Keywords:** Bathymetry, DEM, hydrology, reservoir, satellite remote sensing, TanDEM-X, water resources

## Abstract

Area–volume–elevation (AVE) curves are critical for reservoir operation rules. However, such curves are not publicly available for most global reservoirs. Here, we present a framework to derive reservoir AVE curves from TanDEM-X data, using Lake Mead (~600 km^2^) as an example. First, the maximum water extent from 1984 to 2018—provided by the global surface water (GSW) dataset—was used as a mask to obtain the TanDEM-X data. Then, the TanDEM-X water indication mask (WAM) was applied to the extracted TanDEM-X data to obtain the visible bathymetry, which represents the topography between the maximum extent (according to GSW) and the water extent from WAM. Last, the AVE curve was generated by integrating the volume values from the top to bottom layers. TanDEM-X also captures the elevation values of the transitional waters, which are defined as the difference between the highest and lowest water levels. The transitional waters were obtained by thresholding amplitude and coherence images, and their elevations were then added to the visible bathymetry to extend the AVE curves with an elevation range extending from 344–369 m to 341–369 m. Validation results against *in situ* lidar survey values suggest a high-accuracy of elevation–area (E-A) relationships with *R*^2^ values of >0.99 and NRMSE values from 2.11% to 2.45%, and elevation–volume (E-V) relationships with *R*^2^ values of 1 and NRMSE values from 1.11% to 1.29%. Results also show that TanDEM-X data can capture the interannual variations due to multiple acquisitions, and that the elevation measurements for the lake shore areas are reliable.

## I. Introduction

Four billion people are facing water scarcity [1], and freshwater-related risks are increasing significantly with the earth’s changing climate [2]. Under these circumstances, reservoirs are playing an increasingly important role in the regulation of freshwater and the mitigation of damages—such as hydropower, water supply, irrigation, and flood control. Additionally, reservoirs are essential constituents of the terrestrial system and are deeply involved in thermodynamic, hydrological, and biogeochemical processes [3]–[6]. As the most important metric capturing the physical features of reservoirs, the area–volume–elevation (AVE) curves have been used as critical inputs in modeling these processes [7], [8] although more and more models will require 3-D bathymetry maps in the future [9]. Moreover, the AVE curve can provide useful information related to the monitoring of reservoir storage variations using satellite optical imageries [10]–[15].

Despite its importance, the AVE curve has been oversimplified in many studies due to the limited availability of necessary data from local to global scales. For example, the floodwater depth estimation tool (FwDET) used water surface elevation values from the existing DEM of Lake Houston and found a considerable underestimation over the reservoir area [16]. Moreover, most of the large-scale hydrological models and earth system models (e.g., Community Land Model [17]) assume the constant water surface area and depth, which may contribute to the uncertainty and inconsistency of regional and global simulations [17], [18]. Recently, Yigzaw *et al.* [19] published the first global storage-area-depth dataset for over 6800 reservoirs by appropriating the reservoir geometries. However, results can have relatively large uncertainties and errors, especially for the reservoirs with irregular and complicated shapes. Additionally, they only evaluated the storage–depth relationships, while the performance levels of area–depth relationships were undetermined.

The AVE curves are generally obtained from surveying, simulation, and/or remote sensing-based methods. Surveying-based methods (e.g., lidar survey and sedimentation survey) can provide the most accurate curves. However, it is expensive and labor- and time-intensive—especially for large reservoirs. Simulation-based methods use mathematical equations to derive the AVE curves from the reservoirs’ morphological features, but they are highly restricted by the complexity of reservoir geometry [19], [20]. In recent decades, satellite remote sensing has offered the unprecedented alternative of monitoring reservoirs from space. By pairing up the reservoir area and elevation information obtained from satellite observations, the elevation–area (E–A) relationship can be derived and used to calculate the volume values needed to obtain the AVE relationships [10]–[14], [21]. In general, remote-sensing-based methods require a number of pairs to generate a robust E–A relationship. For example, Gao *et al*. [10] combined Moderate Resolution Imaging Spectroradiometer (MODIS) based reservoir areas from 2000 to 2010 with the available radar altimetry observations (during that same period) to establish E–A relationships for 34 reservoirs—which were then used to estimate the volume variations. More recently, Li *et al.* [13] developed a method that projects lidar tracks onto the water occurrence image—generated from the water classification results of Landsat scenes from 1982 to 2017—to derive the AVE relationships. Although a minimum of one single lidar track is required (for a given reservoir), this technique still relies on long-term Landsat observations. In addition, the process of associating elevation with area values may introduce additional errors because they are collected by different satellite missions with time gaps. Most recently, Li *et al.* [14] generated the AVE relationships for 347 reservoirs at a global scale using multisource satellite altimetry and imagery (though it focused on relatively large reservoirs).

In comparison, topographic data can provide both area and elevation information, and has been an excellent data source for deriving AVE curves [15], [22]–[25]. During the last two decades, the availability of global DEM datasets has significantly increased. These include the Shuttle Radar Topography Mission (SRTM) [26], the National Aeronautics and Space Administration’s DEM (NASADEM) [27], the Advanced Land Observing Satellite (ALOS) Global Digital Surface Model (AW3D30) [28], the Multierror-Removed Improved-Terrain (MERIT) DEM [29], and the Advanced Spaceborne Thermal Emission and Reflection Radiometer (ASTER) Global DEM (GDEM) [30]. The SRTM DEM is the most complete and commonly used global DEM dataset in hydrology [26]. However, the SRTM data were collected within an 11-day period in February 2000 [26]—when many reservoirs experienced high-fill—which makes it difficult to derive AVE curves for these reservoirs.

In this article, we propose a framework to derive reservoir AVE curves using TanDEM-X [31], [32] data. This framework provides the following advantages over the other aforementioned methods: First, the elevations and their associated area values can be obtained simultaneously from one image, which provides high spatiotemporal consistency; and, second, the TanDEM-X dataset is a composite DEM product based on measurements from multiple acquisitions from 2010 to 2015. This can provide more reliable measurements that are less limited by high-level conditions when compared to DEMs collected with a short acquisition phase (such as the SRTM DEM). Furthermore, we adopted the maximum water extent provided by the global surface water (GSW) dataset (based on the Landsat archives from 1984 to 2018), which can better delineate the maximum boundaries of the reservoirs. Validations against lidar surveyed AVE curves suggest a high-level accuracy for this method.

## II. Data and Methods

### A. Study Area and Datasets

Lake Mead (36.25°N, 114.39°W)—formed by the Hoover Dam in September 1935—was selected as the study area. Located on the Colorado River, it is the largest reservoir in the United States in terms of storage. However, the reservoir volume has experienced a significant decrease since 2000 due to drought and increased water demand, with annual mean water levels decreasing from 366.84 (2000) to 328.34 m (2016).

TanDEM-X DEM is a composite data product based on multiple observations from December 2010 to January 2015 collected by two twin synthetic aperture radar (SAR) satellites (i.e., TerraSAR-X and TanDEM-X) [33], [34]. The publicly accessible data with a spatial resolution of 90 m were obtained from the German Aerospace Center (DLR)^1^. The global accuracy of the absolute elevation values is 10 m with a 90% confidence level [35]. In addition to the DEM data, the water indication mask (WAM) [36]—also provided by DLR—can be used to identify the water pixels. Note that, due to the low coherence of the water body in the interferometric data, the water heights are random and may not be meaningful in the TanDEM-X dataset [34]. However, during the TanDEM-X acquisition time, coastal areas (i.e., transitional waters) had multiple states of coverage. Due to the interannual and seasonal water level variations, these pixels were sometimes covered by water while at other times were exposed as land. In the final TanDEM-X DEM product, the elevations observed from all of the acquisitions at a given pixel were weighted to generate a composite value for that pixel. Specifically, the multiple elevations were weighted by Height Error Map (HEM) values in the fusion process, which assures a relatively low (to zero) weight/contribution for the measurements associated with temporary water coverage [34]. Therefore, the elevation values for transitional water pixels are redeemed as reliable.

The maximum water extent of Lake Mead was extracted using the GSW dataset^2^, which is based on the long-term Landsat measurements from 1984 to 2015 [37]. Recently, the GSW dataset was further extended to the year 2018. This represents the maximum extent that water has ever been detected from 1984 to 2018, which can better delineate the water boundaries when compared to those from the HydroLAKES [38] and Global Reservoir and Dam (GRanD) [39] databases—which were mainly derived from various vector datasets and static remote sensing imagery (e.g., SRTM DEM).

The *in situ* AVE curve of Lake Mead was derived from a lidar survey conducted by the United States Bureau of Reclamation (USBR) in 2009, and was used for validation purposes. The lidar survey with 1-m resolution collected detailed elevation values ranging from 333.76 (1095 ft) to 374.90 m (1230 ft). The associated area and volume for each elevation was obtained from the lookup tables provided by USBR [40].

### B. Methods

**Fig. 1.**
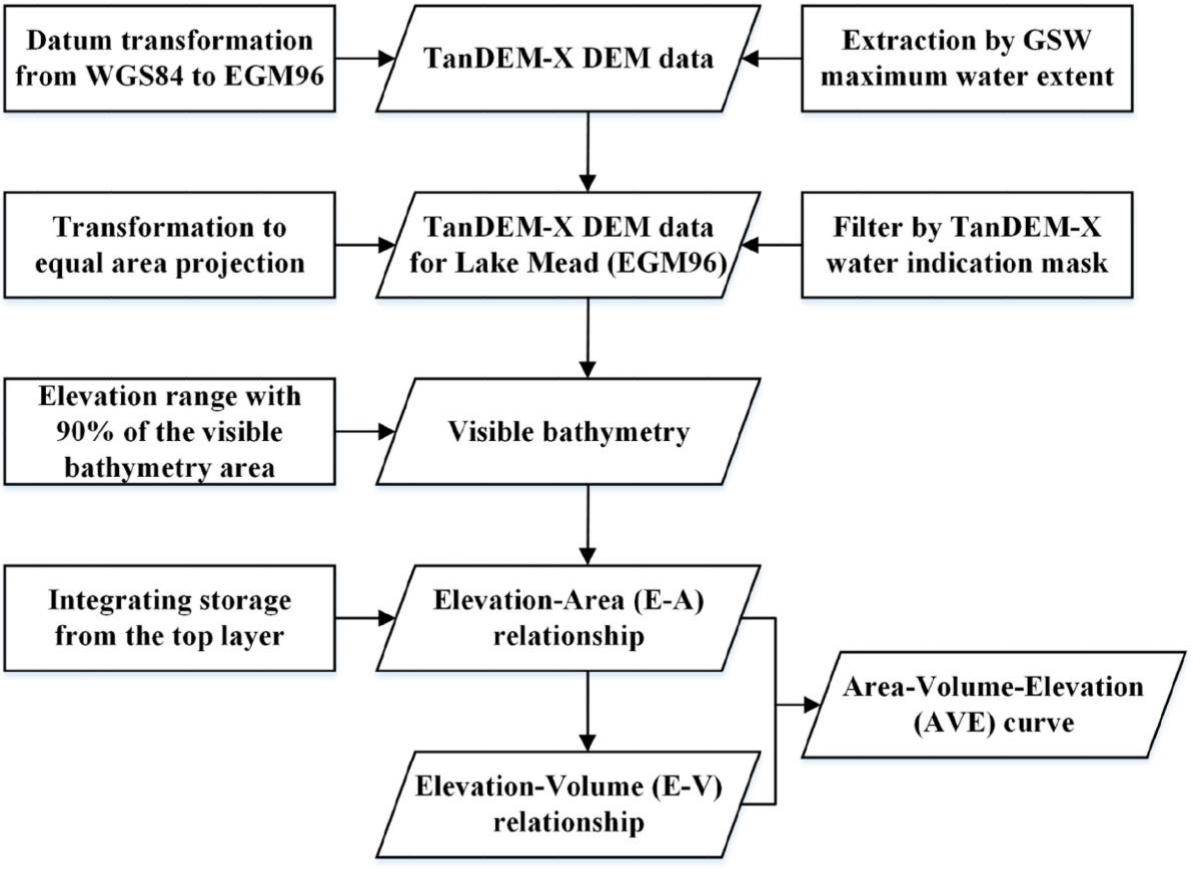
Flowchart for deriving the AVE curve from TanDEM-X data.

The flowchart of this framework for deriving the AVE curve is shown in Fig. 1. First, the TanDEM-X data were transformed from WGS84 ellipsoidal heights to EGM96 orthometric heights using the VDatum tool provided by the National Oceanic and Atmospheric Administration (NOAA)^3^. After this, TanDEM-X elevation values for Lake Mead were extracted using the GSW maximum water extent as a mask, and then the elevation imagery was transformed from GCS_WGS_1984 to an equal-area projection (i.e., World_Cylindrical_Equal_Area) with each pixel representing a 90 by 90 m area. In the next step, the WAM layer was overlaid onto the DEM imagery to mask out the water area and obtain valid elevation values, which represent the visible bathymetry of Lake Mead. The visible bathymetry represents the area between the maximum extent (according to the GSW) and the extent of the water mask (from WAM). All elevations with the same value were taken as a band, and the area associated with each band was calculated. Note that the TanDEM-X values were transformed into integers, and therefore, the elevation bands are at 1-m intervals. The band areas were sorted into a descending order, and the bands associated with the most pixels—which represent 90% of the visible bathymetry area—were used to generate the AVE curve. This helps to eliminate the effects of the extreme elevation values, which only account for a small portion of the overall population of elevation values. Starting from the top band, the area value within each band was calculated by integrating the enclosed pixels, generating the E–A relationship. Last, the volume associated with the *n*^th^ band (*V*_*n*_) was calculated after the following equation:

(1) $$V_{n}=V_{c}\;-\;\sum_{i=0}^{n\;-\;1}\frac{\left(E_{i}\;-\;E_{i+1}\right)\left(A_{i}+A_{i+1}\right)}{2}$$

where *V*_*c*_ is the volume at capacity provided by the USBR [40]. *A*_*i*_ is the entire water surface area enclosed by the *i*^th^ band, which corresponds to the elevation *E*_*i*_. Thus, the volume value corresponding to each band was obtained, and the elevation–volume (E–V) relationship was derived. Finally, the AVE curve was generated by combining the E–A and E–V relationships.

As an important by product of the TanDEM-X dataset, WAM reflects the process of water body detection. Because water areas are generally very incoherent, the water heights are random in the TanDEM-X dataset and may not be meaningful [34]. However, the WAM layer can be used for delineating the coastal waters, and the DEM mosaicking approach can take full advantage of the multiple measurements [41]. Therefore, the transitional waters in lake shore areas may provide additional information for AVE generation. The transitional waters are defined by the difference between the highest and lowest water levels, which represent interannual variations. Depending on the water level, these pixels are sometimes covered by water but other times are exposed as land. WAM uses three criteria to detect water: 1) a strict radar brightness threshold of −18 dB for the SAR amplitude (strict AMP Thresh1); 2) a more relaxed radar brightness threshold of −15 dB for the SAR amplitude (relaxed AMP Thresh2); and 3) a threshold of 0.23 for the interferometric coherence (COH Thresh) [36]. Globally, the accuracy for water bodies detected from amplitude and coherence images are up to 71.3% and 71.7%, respectively [36]. The WAM values are coded in a bit mask, with each bit value representing the number of acquisitions with water detected [34]. The WAM values can be used to derive a binary water mask by thresholding the 0–255 WAM byte values. For example, by selecting WAM values from 3 to 7, a water mask using a relaxed AMP-Thresh2 can be obtained. In this case, we first added the pixels from the water mask of the relaxed AMP-Thresh2 to the visible bathymetry, which was then used to derive a new AVE curve. Then, we sequentially included the water mask using different criteria—the strict AMP-Thresh1 (9 ≤ WAM ≤ 31), 1× water with the COH-Thresh (i.e., water was detected once, 33 ≤ WAM ≤ 63), and 2× water with the COH-Thresh (i.e., water was detected twice, 65 ≤ WAM ≤ 95)—and then generated the corresponding AVE curves. It should be noted that we did not use the criteria defined by 3× water with the COH-Thresh (i.e., water was detected three or more times, WAM ≥ 97) because these pixels primarily refer to the permanent waters with extreme height values that are not meaningful.

## III. Results

### A. Visible Bathymetry Derived From TanDEM-X Data

**Fig. 2.**
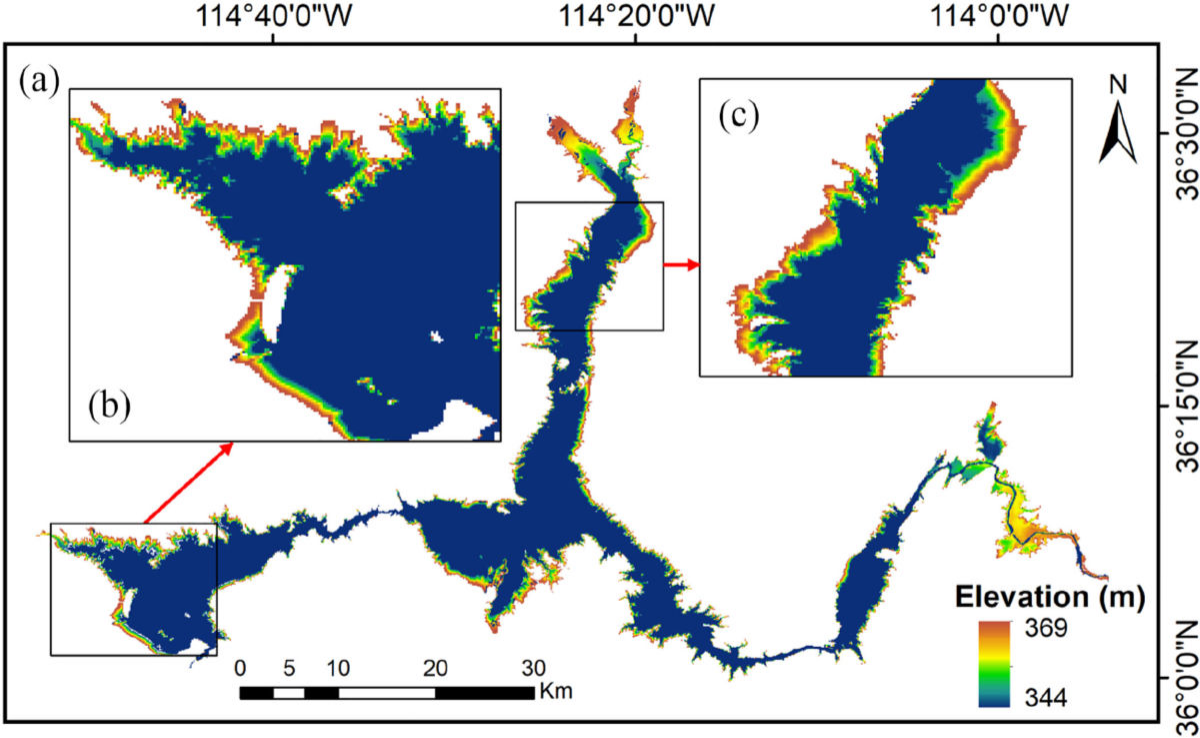
Visible bathymetry from TanDEM-X data used for deriving the AVE curve. (a) Overall pattern. (b)-(c) Close-up views of western and northern regions.

The maximum extent of Lake Mead from GSW is 601.67 km^2^, with valid elevation values (i.e., WAM = 1) ranging from 228.66 to 560.35 m, which cover an area of 193.42 km^2^. The extreme high and low elevation values are primarily attributed to two sources. The first comes from the surrounding pixels that represent mountains, which were misclassified as water during the production of the maximum extent layer. The second source is related to the fact that water bodies have very incoherent areas, combined with the fact that measurements of the pixels near these water bodies may be interfered with. However, these extreme values only account for a small portion of the overall data and were excluded from the AVE generation. The elevations ranging from 344 to 369 m—which represent 90% of the visible bathymetry area—were used to generate the AVE curve. This indicates that the water levels during the TanDEM-X acquisition period (2010–2015) were generally less than 344 m due to the historically extended drought since 1999. This is consistent with *in situ* measured elevation values during this period, which range from 329.37 to 345.70 m. The TanDEM-X data over Lake Mead and its two subregions are shown in Fig. 2. For visualization purposes, the pixels with elevations greater than 369 m were excluded, and the pixels with elevations lower than 344 m (and/or within the WAM area) were assigned a value of 344 m. Overall, the elevations show well-organized patterns and gradients (for example, the shapes of islands were clearly captured).

**Fig. 3.**
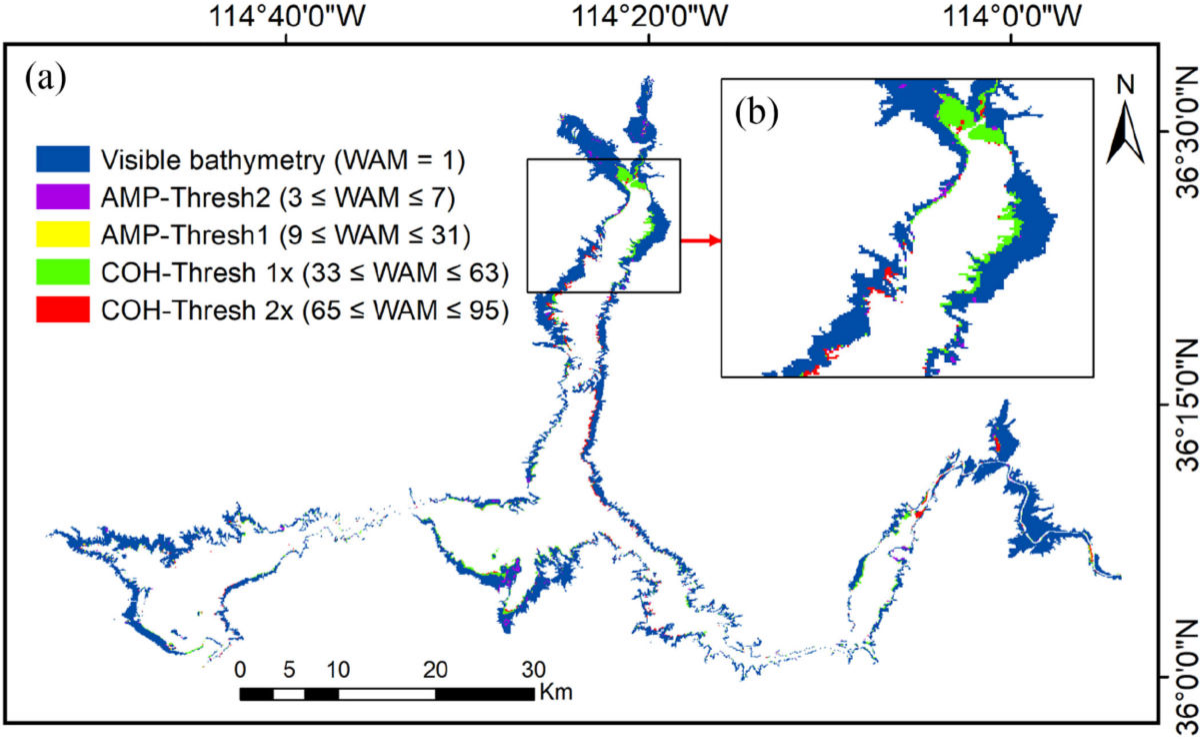
(a) Extent of visible bathymetry and waters detected by different methods with different WAM thresholds. (b) Close-up view focusing on the northern part.

**Fig. 4.**
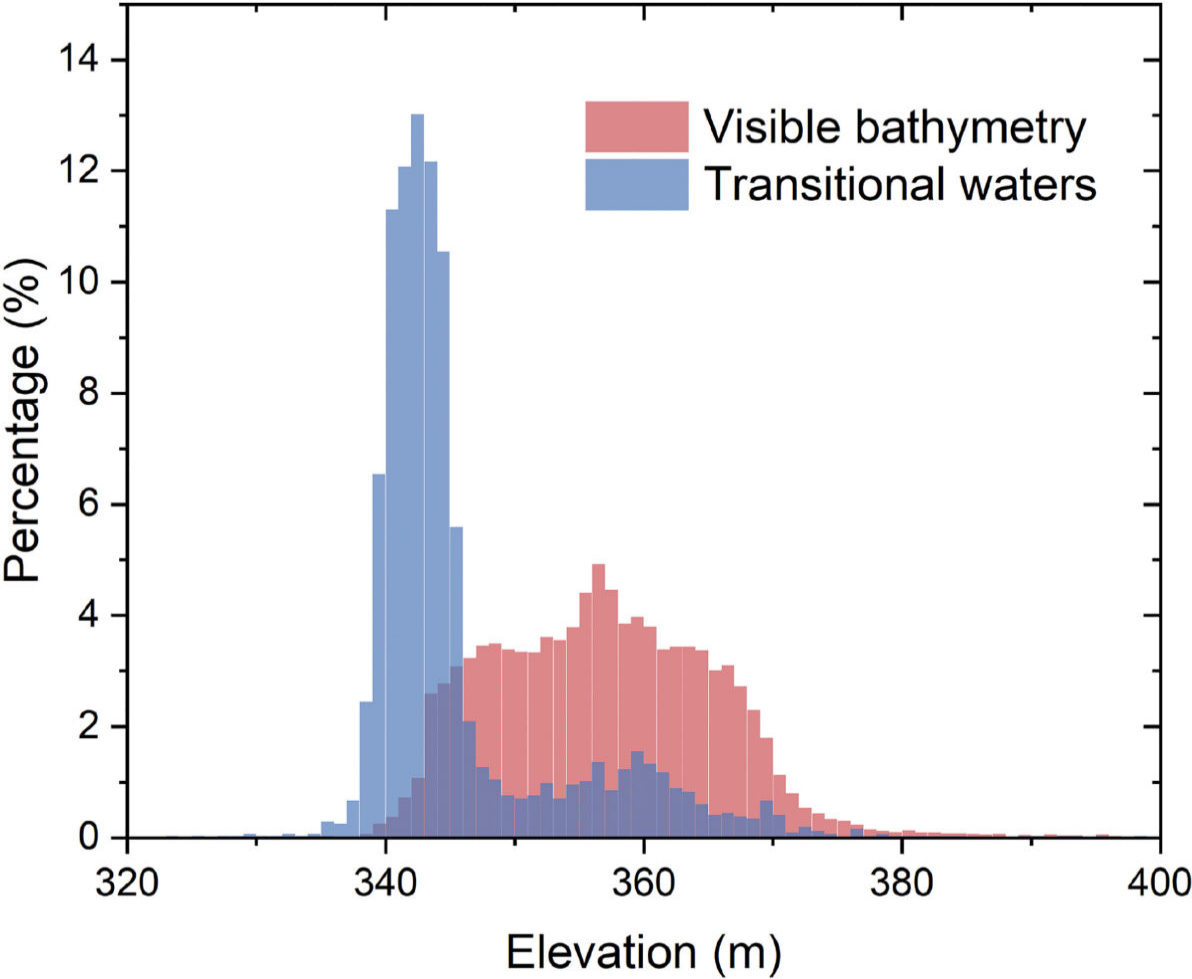
TanDEM-X elevation distributions of visible bathymetry (WAM = 1) and transitional waters (3 ≤ WAM ≤ 95). The transitional waters include all of the pixels detected by AMP-Thresh2, AMP-Thresh1, COH-Thresh 1x, and COH-Thresh 2x.

The transitional waters detected by WAM can extend the coverage of the visible bathymetry. By including the waters detected with the relaxed AMP Thresh2, the area of visible bathymetry has been increased to 198.22 km^2^. Then, after successively adding the water pixels detected with the strict AMP-Thresh1, COH-Thresh with 1x water, and COH-Thresh with 2x water, the areas of visible bathymetry were increased to 198.99,212.36, and 218.93 km^2^, respectively. This indicates that the transitional waters can enhance the visible bathymetry by up to 13% (from 198.22 to 218.93 km^2^). Fig. 3(a) shows the extent of visible bathymetry and waters detected by different methods and different WAM thresholds, with a close-up view focusing on the northern part [see Fig. 3(b)]. It shows that the transitional waters are mainly located along the coastal regions, which represent the interannual variations. Fig. 4 shows the elevation distribution of visible bathymetry and transitional waters. The elevation values of the visible bathymetry have an approximately normal distribution with the peak at the elevation of 357 m. The transitional waters have an elevation range that overlaps with that of the visible bathymetry, but the peak is shifted to a lower elevation value of 342 m. The *in situ* elevation observations during the TanDEM-X data acquisition period range from 329.37 to 345.70 m, with the majority of the transitional waters falling into this range. Note that fewer transitional waters are observed with elevations less than 337 m. This is because we used strict criteria (3 ≤ WAM ≤ 95) to select transitional waters, and those very close to the permanent waters were not selected in this study. Due to the multiple acquisitions—and the mosaicking strategy of TanDEM-X [41]—the transitional waters were assumed to have valid elevation values (depending on the existence of measurements taken when they were not immersed in the water). With regard to the remaining pixels within the reservoir area, they were detected using COH-Thresh 3x (water was detected at least three times, WAM ≥ 97). Most of these are located in permanent waters with nonmeaningful elevation values.

### B. Validation of the AVE Curve Using Lidar Surveys

**Fig. 5.**
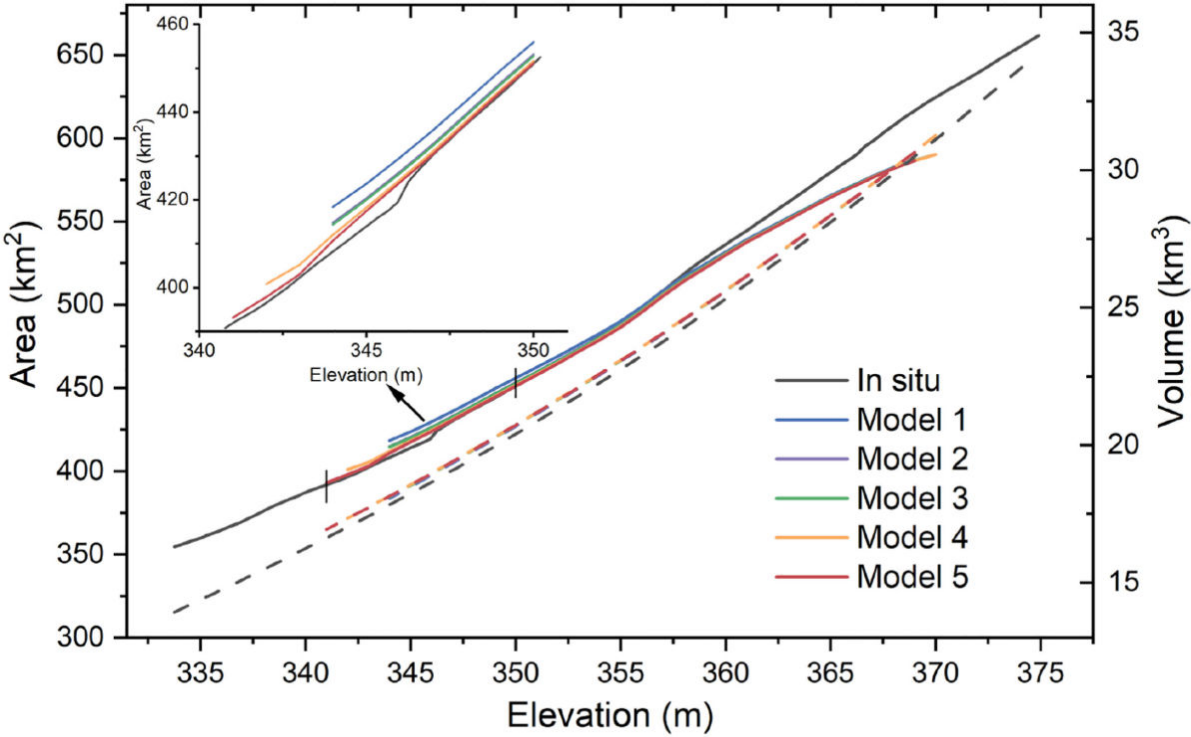
Validation of the AVE curves for Lake Mead derived from TanDEM-X with different WAM thresholds against the lidar surveyed curve. The solid lines represent the E–A curves, and the dashed lines represent the E–V curves. The close-up view shows the E–A curves with elevations from 341 to 350 m. The definitions and validation results of Models 1–5 are summarized in Table I.

**Fig. 6.**
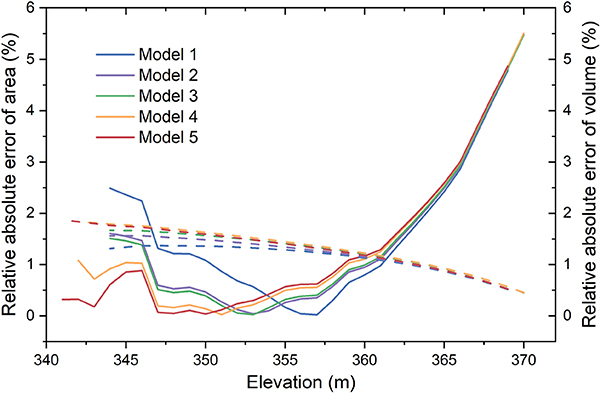
Relative absolute error of area (solid lines) and volume (dashed lines) across the elevation range.

The E–A and E–V relationships were compared with their counterparts from the lidar survey (see Fig. 5), and the validation results show good agreement (see Fig. 6 and Table I). In addition to the AVE curve from the visible bathymetry (Model 1), we also evaluated the curve’s accuracy when including the transitional water pixels with WAM thresholds (Models 2–5). Though the vertical biases are relatively large at high elevations (especially with the elevations over 365 m), the E–A relationship shows good overall consistency with the lidar survey (with NRMSE values ranging from 2.11% to 2.45%). The relatively large area bias can be explained by the difference of maximum reservoir extent between GSW (601.67 km^2^) and USBR (661.68 km^2^). This difference leads to an underestimation of area values from the TanDEM-X-based E–A relationship for the high elevation domain. More importantly, the inclusion of transitional waters can extend the visible bathymetry and improve performance. From the original visible bathymetry, the elevation range used for constructing the AVE curve is from 344 to 369 m. When including the transitional waters, the range is extended to 341–369 m. As shown in the close-up view of the E–A curve in Fig. 5, the derived curves (with elevations below 350 m) are getting closer to the lidar surveyed values with the extension of the transitional waters (from Model 1 to 5). Moreover, for a given elevation value below 350 m, the relative absolute error of the area decreases from Model 1 to 5 (see Fig. 6). However, the inclusion of transitional waters did not improve the accuracy of the E–A relationship when the elevations were over 355 m (see Fig. 6). This indicates that transitional waters with elevation values larger than 355 m are not very reliable (although we note that these unreliable transitional waters only account for a small portion; Fig. 4).

With regard to the E–V relationships, it is evident that they are in very good agreement with the lidar survey (see Figs. 5 and 6)—with NRMSE values from 1.11% to 1.29% (see Table I). It is observed that the discrepancies in the E–A relationships have only small effects on the E–V relationships. The relative absolute errors of the volume values increase as the elevation decreases (see Fig. 6). This is because the storage values are integrated from the top and the errors are accumulated at the bottom. Although the transitional waters did not improve the E–V relationship (due to the large vertical bias for elevations over 355 m), all of the E–V cures show good results, with the highest relative absolute error of the volume value being less than 2% (see Fig. 6).

## IV. Discussion

### A. Comparison With SRTM DEM

SRTM DEM data have been widely used in hydrologic applications [26], [42]–[44]. The linear vertical absolute height error is less than 16 m with a 90% confidence level [26]. In order to compare with the results from the SRTM, we collected SRTM version 3.0 data—provided by JPL/NASA with 30-m resolution, which has been void-filled with open source data—using the same extent of Lake Mead as that from GSW, which is from the Google Earth Engine (GEE) platform [45].

The SRTM elevations over Lake Mead range from 206 to 590 m. Similar to the TanDEM-X data, the extreme high and low values in SRTM are primarily attributed to errors (of the maximum extent layer, and vertical measurements over water). The water level detected by SRTM was 372 m, which represents an area of 571.84 km^2^ and accounts for 98% of the maximum extent (according to GSW). This suggests that the visible bathymetry that can be used for AVE generation from SRTM DEM represents less than 2% of the total area. More importantly, no elevation gradients or patterns can be found from the SRTM data, and the elevations from 372 to 375 represent 99% of the total area. Therefore, it is not feasible to generate a reliable AVE curve from SRTM DEM for Lake Mead. With regard to the TanDEM-X data, the visible bathymetry accounts for 32% of the total area, which shows clear patterns of gradients.

**Fig. 7.**
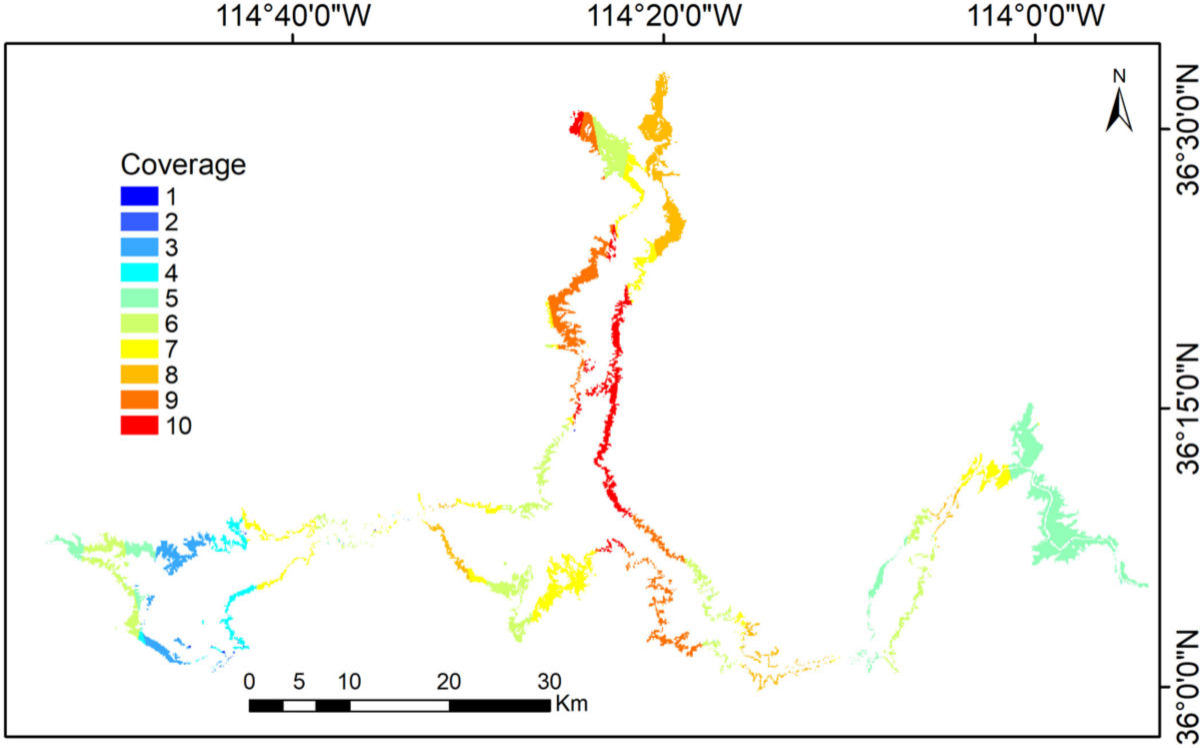
Coverage map (COV) for the visible bathymetry over Lake Mead from December 2010 to January 2015.

It is apparent that SRTM provided less bathymetry information compared to TanDEM-X data. This is because Lake Mead was at a relatively high level during the acquisition time of SRTM DEM. According to the elevation gage at Hoover Dam, the *in situ* value was 369.96 m at the end of February 2000^4^. However, the TanDEM-X data were composited based on multiple acquisitions from December 2010 to January 2015, which captured the low-fill times of the reservoir (with *in situ* elevations ranging from 329.37 to 345.70 m). According to the Coverage map (COV) layer—a product provided by DLR to indicate the number of valid elevation observations that were available for compositing—the maximum COV value for visible bathymetry of Lake Mead is 10, while the median value is 7 (see Fig. 7). The TanDEM-X product was composited by means of a weighted average (with the height error used as the weight), which takes advantage of multiple observations to reduce the uncertainty and improve the accuracy [41].

It is noteworthy that the WAM of TanDEM-X can capture the interannual variations, which increased the area of visible bathymetry of Lake Mead by up to 13%. However, the primary reason that the TanDEM-X data was more appropriate to derive the AVE curve is that they were collected during a period when Lake Mead was in a low fill state. Additionally, the multiple acquisitions (of TanDEM-X) have a better chance to capture the low fill occurrences. According to Zhao and Gao [46], the area of Lake Mead was 579.19 km^2^ in February 2000 when SRTM DEM data were collected. During the TanDEM-X acquisition period (December 2010 to January 2015), the corresponding areas varied from 338.88 to 405.46 km^2^, which provides 30%–41% more bathymetry information as compared to SRTM DEM. Furthermore, Gesch [47] recently reported that TanDEM-X performs the best in delineating the contours of the low-elevation coastal zone (with an RMSE of 1.69 m) when compared to other global DEM datasets, including NASADEM (RMSE = 3.10 m), ALOS AW3D30 (RMSE = 3.12 m), MERIT (RMSE = 3.14 m), SRTM (RMSE = 5.57 m), and ASTER GDEM (RMSE = 9.47 m). However, this result was generated from a more highly processed, edited version of TanDEM-X, with a spatial resolution of 12 m—which is available as a commercial product, WorldDEM. With regard to the publicly accessible, 90-m resolution version, the elevations of the water bodies are not edited and the values may not be valid.

### B. Potential Applications

**Fig. 8.**
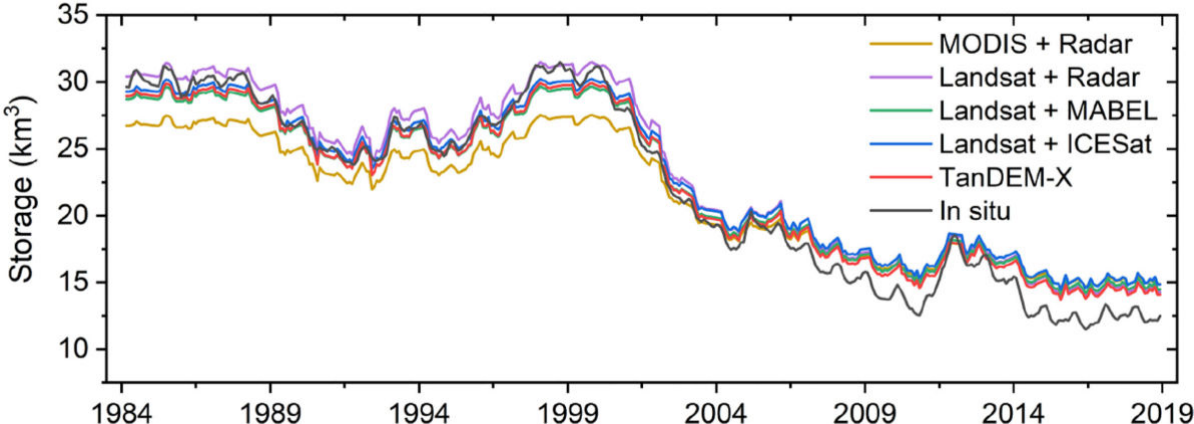
Storage estimations of Lake Mead from 1984 to 2018 using different E–A relationships. The E–A relationships were derived from different datasets (e.g., radar altimetry-based elevation measurements and MODIS-based area estimations). The time-series monthly surface area of Lake Mead provided by Zhao and Gao [46] was applied to these E–A relationships to obtain the corresponding storage values.

The derived AVE curve can help to improve various water-resources-related studies and applications. Previous studies have shown that the quality of the E–A relationships has a significant impact on the accuracy of the remotely sensed reservoir storages [48]. We applied the E–A relationships from different studies to the same monthly water area values for Lake Mead derived from Landsat observations between 1984 and 2018 [46] to assess their performances. Validation results show that the remotely sensed water areas are in good agreement with *in situ* observations [46]. The monthly gauge elevations provided by the USBR^4^ were applied to the lidar surveyed E–V curves [40] to obtain *in situ* storage values. The storage estimates using different E–A relationships are shown in Fig. 8, with the statistics summarized in Table II. The E–A relationship from Gao *et al*. [10]—derived from the MODIS-based water area and radar altimetry data—has the lowest *R*^2^ value (0.69), and the largest RMSE of the storage estimations (2.30 km^3^). This is primarily due to the low resolution of MODIS data (250 m) and the limitation of the classification algorithm with regard to handling image contaminations [10]. By replacing the MODIS data with Landsat, Duan and Bastiaanssen [11] improved the *R*^2^ and RMSE values to 0.99 and 1.31 km^3^, respectively. Li *et al*. [13] developed a novel algorithm by projecting a single photon-counting lidar track onto the water occurrence image. The lidar data were collected by the Multiple Altimeter Beam Experimental Lidar (MABEL) instrument—the prototype of ICESat-2—and the occurrence percentile image was obtained by stacking the long-term Landsat water classifications from 1982 to 2017. This method reduced the RMSE to 1.10 km^3^. More recently, Li *et al*. [14] extended this method to ICESat data, which also shows fairly good performance with an RMSE value of 1.21 km^3^. However, the results from MABEL are slightly better due to its higher spatial resolution and vertical accuracy. Overall, the storage estimations using lidar altimeter (i.e., MABEL and ICESat) based E–A relationships outperformed the radar altimetry-based methods. This is due to the fact that lidar instruments can provide more accurate elevation measurements. The E–A relationship derived from TanDEM-X outperformed the abovementioned studies in terms of *R*^2^ (*>*0.99) and RMSE (0.92 km^3^). This is attributed to the fact that TanDEM-X can simultaneously provide the elevation and area information, which reduces the uncertainties from pairing up elevation and area values that are from different sources. Moreover, the TanDEM-X product is composited from multiple observations, which improves vertical accuracy and reduces the random elevation error. The storage estimations using the E–A relationships from different WAM thresholds (see Fig. 5) also show good results, with RMSE values ranging from 1.01 to 1.07 km^3^.

Remotely sensed storage variations can provide useful information for many aspects of reservoir management. The storage values are directly related to water availability, which can support the decision-making process in matters related to water supply and flood control. Moreover, a hydrological drought index can be developed based on storage estimates. Recently, Zhao and Gao [49] introduced a framework to monitor hydrological droughts using a reservoir surface area dataset. Reservoir storage should be a better indicator than the surface area in evaluating hydrological droughts.

### C. Limitations and Future Directions

Despite the high accuracy of the AVE curve from TanDEM-X data, this method still has some limitations that should be addressed. First, it cannot generate the AVE relationships within the entire elevation range, except in cases when the reservoirs were mostly empty or not yet constructed during the acquisition time of the TanDEM-X data. The full AVE curve can be obtained by extrapolation, which may have large uncertainty. The accuracy and representation of the partial curve derived from the DEM determine the quality of the extrapolated part. Second, it is not applicable to reservoirs with high water levels and small variations during the acquisition period. However, reservoirs are designed for multiple purposes (e.g., water supply, irrigation, and hydropower generation), and are highly impacted by human activities that tend to experience large seasonal and interannual variations. Therefore, TanDEM-X has the potential to be applied to numerous reservoirs—especially those that experienced low levels, and/or large dynamics, during the data acquisition period (2010–2015). Meanwhile, with regard to the reservoirs that remained at high water levels, a sedimentation survey is the most reliable means to obtain the underwater topography. Third, this method was developed over one single reservoir. We specifically used an elevation range, which accounts for 90% of the visible bathymetry area in Lake Mead. However, this threshold value may vary with the reservoir being studied, and further investigation should be conducted to evaluate its representativeness. Li *et al*. [14] used a Landsat-based method (with a spatial resolution of 30 m) to derive the bathymetry maps and AVE curves for global reservoirs, with the smallest reservoir included being 1.47 km^2^. Due to the 90-m spatial resolution of TanDEM-X data, this method should be applicable to reservoirs with areas larger than 10 km^2^. Additionally, the commercial TanDEM-X product—which has a spatial resolution of 12 m—can observe much smaller reservoirs.

In general, global DEM datasets were collected at various phases and correspond to different water levels for a given reservoir. Therefore, a global dataset of reservoir AVE curves from a single DEM dataset is limited by the visible bathymetry. The most appropriate strategy is to combine all of the available DEM datasets to achieve the maximum coverage of global reservoirs. This could lead to a good complement to the AVE curves derived from the Global Reservoir Bathymetry Dataset (GRBD) [14], which will improve the modeling of reservoirs in land surface and earth system models. Additionally, each DEM dataset can be used to derive the full AVE curve for reservoirs built after the data acquisition period. For example, the SRTM DEM dataset can cover those constructed after February 2000. It has been reported that reservoir construction activities are still ongoing at a high rate in the 21st century (especially in developing countries), with 3700 hydropower dams either planned or under construction [50]. With regard to the recently built reservoirs, their AVE curves can be accurately retrieved from the currently available DEM datasets.

## V. Conclusion

In this article, we proposed a framework to derive reservoir AVE curves using TanDEM-X data. With multiple acquisitions collected for each pixel, the composite TanDEM-X product can offer much better reservoir bathymetry coverage than other single acquisition-based DEM datasets (e.g., SRTM). The visible bathymetry from TanDEM-X shows clear patterns and gradients for Lake Mead (see Fig. 2). Based on the visible bathymetry (from 344 to 369 m), we derived the AVE curve for Lake Mead, which agrees well with the lidar survey values. The NRMSE values of the E–A and E–V relationships are 2.19% and 1.11%, respectively. Additionally, benefiting from the interannual variations captured by multiple acquisitions, the transitional waters can also be detected by TanDEM-X DEM. By including the transitional waters, the visible bathymetry can be extended to an elevation range of 341–369 m, with an effective area increase of 13%, and an RMSE value for the E–A relationship reduced by 6.12% (see Fig. 5 and Table I). This suggests that the elevation measurements for transitional waters are reliable in the TanDEM-X dataset. High-quality measurements of these waters can extend the visible bathymetry and improve the quality of the AVE curves derived for reservoirs that experienced large variations during the TanDEM-X data collection phase (2010–2015). Moreover, we compared the storage estimations for Lake Mead from 1984 to 2018 using E–A relationships from different studies. Results show that our E–A relationship has the best performance in terms of RMSE (see Fig. 8 and Table II).

It should be noted that Lake Mead has been continuously shrinking since 2000 due to severe droughts and increasing water demand. Changing rainfall patterns and current water use patterns are putting pressure on water resources management at Lake Mead, as the population relying on it for water usage continues to increase. The AVE curve has become an important indicator for supporting spatial decision-making in water resources management activities such as optimizing water harvesting and urban water consumption. The results indicate that TanDEM-X was able to capture a large amount of visible bathymetry during the acquisition time when Lake Mead experienced low water levels, with observed elevations from 329.37 to 345.70 m. Regarding the SRTM DEM dataset, it was collected in February 2000 when Lake Mead was at a high level (369.96 m)—so only a small domain of visible bathymetry was obtained, which is not adequate for generating the AVE curve. The values contained in global DEM datasets were acquired at different times and are associated with various extents of the visible bathymetry for each reservoir. Thus, a global AVE dataset can be generated by combining all of the available DEM datasets. This dataset will complement the AVE curves derived from the GRBD [14]. The E–A relationships can further extend the coverage of GRBD, and can also update the global reservoir storage-area-depth dataset [19]—which has large uncertainties due to the limitations of mathematical approximation, and the complexities of reservoir geometry. While this method has been specifically examined using Lake Mead, future work will focus on extending this method to global reservoirs to investigate its applicability.

## Figures and Tables

**TABLE I:** Summary of AVE Curves for Lake Mead Derived From TanDEM-X With Different WAM Thresholds

Model	Extent	Elevation range (m)	*n*	Visible percent	R^2^ (E-A)	R^2^ (E-V)	RMSE_A (km^2^)	NRMSE_A (%)	RMSE_V (km^3^)	NRMSE_V (%)
1	Visible bathymetry (WAM = 1)	344–369	26	32.15%	>0.99	1	11.11	2.19	0.26	1.11
2	Model 1 + AMP-Thresh2 (3≤ WAM ≤ 7)	344–369	26	32.95%	>0.99	1	10.82	2.13	0.28	1.18
3	Model 2 + AMP-Threshl (9 ≤ WAM ≤ 31)	344–370	27	33.07%	>0.99	1	12.53	2.45	0.29	1.21
4	Model 3 + COH-Thresh l × (33 ≤ WAM ≤ 63)	342–370	29	35.29%	>0.99	1	12.22	2.43	0.30	1.27
5	Model 4 + COH-Thresh 2 × 65 ≤ WAM ≤ 95	341–369	29	36.39%	>0.99	1	10.43	2.11	0.30	1.29

Note that Visible percent represents the percentage of visible bathymetry area to the total area (i.e., the maximum extent from the GSW dataset). RMSE_A and RMSE_V refer to the RMSE values in terms of area and volume in the validation of the E-A and E-V curves. NRMSE_A and NRMSE_V are the normalized RMSE values using the mean *in situ* area and volume measurements, respectively.

**TABLE II:** Summary of the Storage Estimations Using Different E–A Relationships

Model	Area source	Elevation source	*n*	R^2^ of E-A	RMSE (km^3^)
Gao et al. (2012)	MODIS	Radar altimetry	107	0.69	2.30
Duan and Bastiaanssen (2013)	Landsat	Radar altimetry	11	0.99	1.31
Li et al. (2019)	Landsat	MABEL	13	0.98	1.10
Li et al. (2020)	Landsat	ICESat	42	0.99	1.21
TanDEM-X (visible bathymetry)	TanDEM-X	TanDEM-X	26	>0.99	0.92

## Footnotes

1 [Online]. Available: https://download.geoservice.dlr.de/TDM90

2 [Online]. Available: https://global-surface-water.appspot.com

3 [Online]. Available: https://vdatum.noaa.gov/

4 [Online]. Available: https://www.usbr.gov/lc/region/g4000/hourly/meadelv.html, accessed on September 1st, 2020

## References

[1] Mekonnen MM and Hoekstra AY, “Four billion people facing severe water scarcity,” Sci. Adv, vol. 2, no. 2, 2016, Art. no. e1500323. 26933676 10.1126/sciadv.1500323 PMC4758739

[2] Cisneros J, “Freshwater resources,” in Climate Change 2014–Impacts, Adaptation and Vulnerability: Part A: Global and Sectoral Aspects. Working Group II Contribution to the IPCC Fifth Assessment Report. Cambridge, U.K.: Cambridge Univ. Press, 2014, pp. 229–270.

[3] Tranvik LJ , “Lakes and reservoirs as regulators of carbon cycling andclimate,”Limnol.Oceanogr,vol. 54, no. 6part2, pp. 2298–2314, 2009.

[4] Li HY , “Modeling stream temperature in the anthropocene: An earth system modeling approach,” J. Adv. Model. Earth Syst, vol. 7, no. 4, pp. 1661–1679, 2015.

[5] Maavara T , “Global phosphorus retention by river damming,” Proc. Nat. Acad. Sci, vol. 112, no. 51, pp. 15603–15608, 2015. 26644553 10.1073/pnas.1511797112 PMC4697372

[6] Biemans H , “Impact of reservoirs on river discharge and irrigation water supply during the 20th century,” Water Resour. Res, vol. 47, no. 3, pp. 1–15, 2011.

[7] Kerimoglu O and Rinke K, “Stratification dynamics in a shallow reservoir under different hydro-meteorological scenarios and operational strategies,” Water Resour. Res, vol. 49, no. 11, pp. 7518–7527, 2013.

[8] Bowling LC and Lettenmaier DP, “Modeling the effects of lakes and wetlands on the water balance of Arctic environments,” J. Hydrometeorol, vol. 11, no. 2, pp. 276–295, 2010.

[9] Getirana A, Jung HC, Van Den Hoek J, and Ndehedehe CE, “Hydropower dam operation strongly controls lake Victoria’s freshwater storage variability,” Sci. Total Environ, vol. 726, 2020, Art. no. 138343. 32315844 10.1016/j.scitotenv.2020.138343

[10] Gao H, Birkett C, and Lettenmaier DP, “Global monitoring of large reservoir storage from satellite remote sensing,” Water Resour. Res, vol. 48, no. 9, pp. 1–12, 2012.

[11] Duan Z and Bastiaanssen W, “Estimating water volume variations in lakes and reservoirs from four operational satellite altimetry databases and satellite imagery data,” Remote Sens. Environ, vol. 134, pp. 403–416, 2013.

[12] Zhang S, Gao H, and Naz BS, “Monitoring reservoir storage in South Asia from multisatellite remote sensing,” Water Resour. Res, vol. 50, no. 11, pp. 8927–8943, 2014.

[13] Li Y, Gao H, Jasinski MF, Zhang S, and Stoll JD, “Deriving high-resolution reservoir bathymetry from ICES at-2 prototype photon-counting Lidar and Landsat imagery,” IEEE Trans. Geosci. Remote Sens, vol. 57, no. 10, pp. 7883–7893, 10. 2019.

[14] Li Y, Gao H, Zhao G, and Tseng K-H, “A high-resolution bathymetry dataset for global reservoirs using multi-source satellite imagery and altimetry,” Remote Sens. Environ, vol. 244, 2020, Art. no. 111831.

[15] Zhang S and Gao H, “Using the digital elevation model (DEM) to improve the spatial coverage of the MODIS based reservoir monitoring network in South Asia,” Remote Sens, vol. 12, no. 5, pp. 745–760, 2020.

[16] Cohen S , “Estimating flood water depths from flood inundation maps and topography,” JAWRA J. Amer. Water Resour. Assoc, vol. 54, no. 4, pp. 847–858, 2017.

[17] Oleson KW , “Technical description of version 4.0 of the community land model (CLM),” NCAR, Boulder, CO, USA, Tech. Rep NCAR/TN–478+STR, 4. 2010.

[18] Hanasaki N, Kanae S, and Oki T, “A reservoir operation scheme for global river routing models,” J. Hydrol, vol. 327, no. 1–2, pp. 22–41, 2006.

[19] Yigzaw W , “A new global storage-area-depth dataset for modeling reservoirs in land surface and earth system models,” Water Resour. Res, vol. 54, no. 12, pp. 10,372–10,386, 2018.

[20] Johansson H, Brolin AA, and Håkanson L, “New approaches to the modelling of lake basin morphometry,” Environ. Model. Assessment, vol. 12, no. 3, pp. 213–228, 2007.

[21] Getirana A, Jung HC, and Tseng K-H, “Deriving three dimensional reservoir bathymetry from multi-satellite datasets, ”Remote Sens. Environ, vol. 217, pp. 366–374, 2018.

[22] Magome J, Ishidaira H, and Takeuchi K, “Method for satellite monitoring of water storage in reservoirs for efficient regional water management,” Int. Assoc. Hydrological Sci., Publication, vol. 281, pp. 303–310, 2003.

[23] Sayl KN, Muhammad NS, and El-Shafie A, “Optimization of area–volume–elevation curve using GIS–SRTM method for rainwater harvesting in arid areas,” Environ. Earth Sci, vol. 76, no. 10, 2017, Art. no. 368.

[24] Tseng K-H, Shum C, Kim J-W, Wang X, Zhu K, and Cheng X, “Integrating Landsat imageries and digital elevation models to infer water level change in Hoover dam,” IEEE J. Sel. Topics Appl. Earth Observ. Remote Sens, vol. 9, no. 4, pp. 1696–1709, 4. 2016.

[25] Zhang S, Foerster S, Medeiros P, de Araújo JC, Motagh M, and Waske B, “Bathymetric survey of water reservoirs in north-eastern Brazil based on TanDEM-X satellite data,” Sci. Total Environ, vol. 571, pp. 575–593, 2016. 27418521 10.1016/j.scitotenv.2016.07.024

[26] Farr TG , “The shuttle radar topography mission,” Rev. Geophys, vol. 45, no. 2, pp. 1–33, 2007.

[27] Crippen R , “NASADEM global elevation model: Methods and progress,” JPL, Pasadena, CA, USA, Tech. Rep. 2016.

[28] Tadono T , “Generation of the 30M-mesh global digital surface model by ALOS PRISM,” Int. Arch. Photogrammetry, Remote Sens. Spatial Inf. Sci, vol. 41, pp. 157–162, 2016.

[29] Yamazaki D , “A high-accuracy map of global terrain elevations,” Geophysical Res. Lett, vol. 44, no. 11, pp. 5844–5853, 2017.

[30] Abrams M, Bailey B, Tsu H, and Hato M, “The ASTER global DEM,” Photogrammetric Eng. Remote Sens, vol. 76, no. 4, pp. 344–348, 2010.

[31] Krieger G , “TanDEM-X: A satellite formation for high-resolution SAR interferometry,” IEEE Trans. Geosci. Remote Sens, vol. 45, no. 11, pp. 3317–3341, 11. 2007.

[32] Zink M , “TanDEM-X: The new global DEM takes shape,” IEEE Geosci. Remote Sens. Mag, vol. 2, no. 2, pp. 8–23, 6. 2014.

[33] Rizzoli P , “Generation and performance assessment of the global TanDEM-X digital elevation model,” ISPRS-J. Photogramm. Remote Sens, vol. 132, pp. 119–139, 2017.

[34] Wessel B, “TanDEM-X ground segment—DEM products specification document,” DLR, Tech. Rep. TD-GS-PS-0021, 5 2018.

[35] Wessel B, Huber M, Wohlfart C, Marschalk U, Kosmann D, and Roth A, “Accuracy assessment of the global TanDEM-X digital elevation model with GPS data,” ISPRS-J. Photogramm. Remote Sens, vol. 139, pp. 171–182, 2018.

[36] Wendleder A, Wessel B, Roth A, Breunig M, Martin K, and Wagenbrenner S, “TanDEM-X water indication mask: Generation and first evaluation results,” IEEE J. Sel. Topics Appl. Earth Observ. Remote Sens, vol. 6, no. 1, pp. 171–179, 2. 2012.

[37] Pekel J-F, Cottam A, Gorelick N, and Belward AS, “High-resolution mapping of global surface water and its long-term changes,” Nature, vol. 540, no. 7633, pp. 418–422, 2016. 27926733 10.1038/nature20584

[38] Messager ML, Lehner B, Grill G, Nedeva I, and Schmitt O, “Estimating the volume and age of water stored in global lakes using a geo-statistical approach,” Nat. Commun, vol. 7, 2016, Art. no. 13603. 27976671 10.1038/ncomms13603 PMC5171767

[39] Lehner B , “High-resolution mapping of the world’s reservoirs and dams for sustainable river-flow management,” Frontiers Ecol. Environ, vol. 9, no. 9, pp. 494–502, 2011.

[40] United Sates Bureau of Reclamation (USBR), “2009 Lake mead Li-DAR survey,” 2011. [Online]. Available: https://www.usbr.gov/lc/region/g2000/2009MeadLIDARsurvey.pdf

[41] Gruber A, Wessel B, Martone M, and Roth A, “The TanDEM-X DEM mosaicking: Fusion of multiple acquisitions using InSAR quality parameters,” IEEE J. Sel. Topics Appl. Earth Observ. Remote Sens, vol. 9, no. 3, pp. 1047–1057, 3. 2015.

[42] Yang L, Meng X, and Zhang X, “SRTM DEM and its application advances,” Int. J. Remote Sens, vol. 32, no. 14, pp. 3875–3896, 2011.

[43] Ludwig R and Schneider P, “Validation of digital elevation models from SRTM X-SAR for applications in hydrologic modeling,” ISPRS-J. Photogrammetry Remote Sens, vol. 60, no. 5, pp. 339–358, 2006.

[44] Li J and Wong DW, “Effects of DEM sources on hydrologic applications,” Comput., Environ. Urban Syst, vol. 34, no. 3, pp. 251–261, 2010.

[45] Gorelick N, Hancher M, Dixon M, Ilyushchenko S, Thau D, and Moore R, “Google earth engine: Planetary-scale geospatial analysis for everyone,” Remote Sens. Environ, vol. 202, pp. 18–27, 2017.

[46] Zhao G and Gao H, “Automatic correction of contaminated images for assessment of reservoir surface area dynamics,” Geophysical Res. Lett, vol. 45, no. 12, pp. 6092–6099, 2018. 10.1029/2018gl078343 PMC8793793 35095126

[47] Gesch DB, “Best practices for elevation-based assessments of sea-level rise and coastal flooding exposure,” Frontiers Earth Sci., Original Res, vol. 6, no. 230, pp. 1–19, 2018.

[48] Gao H, “Satellite remote sensing of large lakes and reservoirs: From elevation and area to storage,” Wiley Interdisciplinary Rev., Water, vol. 2, no. 2, pp. 147–157, 2015.

[49] Zhao G and Gao H, “Towards global hydrological drought monitoring using remotely sensed reservoir surface area,” Geophysical Res. Lett, vol. 46, no. 22, pp. 13027–13035, 2019.

[50] Zarfl C, Lumsdon AE, Berlekamp J, Tydecks L, and Tockner K, “A global boom in hydropower dam construction,” Aquatic Sci, vol. 77, no. 1, pp. 161–170, 2015.

